# Impact of Chronic Kidney Disease on Clinical, Laboratory, and Echocardiographic Features in Patients with Chronic Heart Failure

**DOI:** 10.3390/diseases14010035

**Published:** 2026-01-20

**Authors:** Anastasija Ilić, Olivera Kovačević, Aleksandra Milovančev, Nikola Mladenović, Dragica Andrić, Dragana Dabović, Milana Jaraković, Srdjan Maletin, Teodora Pantić, Branislav Crnomarković, Mihaela Preveden, Ranko Zdravković, Anastazija Stojšić Milosavljević, Aleksandra Ilić, Lazar Velicki, Andrej Preveden

**Affiliations:** 1 Faculty of Medicine, University of Novi Sad, 21000 Novi Sad, Serbia; 2 Institute of Cardiovascular Diseases Vojvodina, 21204 Sremska Kamenica, Serbia; 3 Faculty of Sport and physical education, University of Novi Sad, 21000 Novi Sad, Serbia

**Keywords:** chronic heart failure, chronic kidney disease, left ventricular remodeling, NT-proBNP, diastolic dysfunction

## Abstract

Objective: The aim of this study was to evaluate the impact of chronic kidney disease (CKD) on clinical presentation, laboratory parameters, ECG, and echocardiographic features of patients with chronic heart failure (CHF). Methods: This retrospective cross-sectional study included 2227 patients hospitalized in a tertiary care center due to CHF. Patients were divided into two groups based on the presence of CKD, defined as eGFR < 60 mL/min/1.73 m^2^. Demographic, clinical, laboratory, and echocardiographic data were collected for all patients. Comparative analyses were performed to assess differences in cardiovascular risk factors, comorbidities, laboratory parameters, and echocardiographic findings between the two groups. Results: The proportion of men was significantly higher in the non-CKD group, whereas women predominated in the CKD group (*p* < 0.001). Dyspnea, orthopnea, leg swelling, claudication, and expectoration were significantly more frequent in patients with CKD, while chest pain and palpitations were more common in the non-CKD group (all *p* < 0.05). A significant difference in the distribution of NYHA functional classes was observed between the groups (*p* < 0.001), with NYHA class IV being more prevalent in the CKD group and classes II and III more frequent in the non-CKD group. Levels of CRP and NT-proBNP were significantly higher in the CKD group (*p* < 0.001). In-hospital mortality was 2.5-fold higher in patients with CKD (28.6% vs. 11.1%; *p* < 0.001). Conclusions: Coexistence of CKD was associated with a more severe clinical presentation, advanced functional limitation, and a distinct laboratory and echocardiographic profile in CHF patients.

## 1. Introduction

Chronic heart failure (CHF) and chronic kidney disease (CKD) are major global health problems, profoundly affecting patients’ quality of life, hospitalization rates, and survival. These conditions are tightly interconnected, with dysfunction in one organ often triggering or exacerbating pathology in the other, a relationship known as cardiorenal syndrome (CRS). CRS reflects a complex interplay of hemodynamic, neurohumoral, and metabolic mechanisms: impaired kidney function can cause fluid and electrolyte disturbances, uremia, and acidosis, which further compromise cardiac function, while heart failure reduces renal perfusion and activates neurohumoral pathways, including the sympathetic nervous system and renin–angiotensin–aldosterone system (RAAS) [1,2,3,4]. Recent molecular-level reviews emphasize that CRS progression is driven by RAAS overactivation, systemic inflammation, oxidative stress, endothelial dysfunction, and tissue fibrosis [5].

CRS is classified into five subtypes based on mechanisms of disease onset and progression. CRS type 1, acute cardiorenal syndrome, occurs when sudden heart dysfunction causes acute kidney injury. CRS type 2, chronic cardiorenal syndrome, develops when CHF leads to gradual renal disfunction. CRS type 3, acute renocardiac syndrome, occurs when acute kidney injury causes acute cardiac disfunction. CRS type 4, chronic renocardiac syndrome, develops when CKD leads to the development or progression of CHF. CRS type 5, secondary (systemic) cardiorenal syndrome, involves systemic disorders, such as sepsis or diabetes, that simultaneously affect both organs, the heart and kidneys [1,2,3,4,6].

CHF and CKD share multiple risk factors, including age, hypertension, and coronary artery disease, and their coexistence is associated with poorer prognosis and higher mortality than either condition alone [7,8,9,10]. Pharmacologic interventions targeting one organ may inadvertently impair the other, emphasizing the need for careful therapeutic selection [4]. Despite their high prevalence and the extensive evidence on their individual impacts, the mechanisms underlying the bidirectional heart–kidney interaction and the factors driving CRS remain incompletely understood. A better understanding of these interactions is essential for optimizing clinical management and risk stratification in patients with chronic cardiac and renal dysfunction.

The aim of this study was to determine the impact of CKD on the demographic, clinical, laboratory, electrocardiographic, and echocardiographic characteristics of patients with CHF, providing insight into the interplay between these two conditions.

## 2. Materials and Methods

This retrospective observational cross-sectional study included patients hospitalized in a tertiary care center due to CHF. Adult patients (≥18 years old) with heart failure were identified using the International Classification of Diseases, 10th edition (ICD-10) diagnostic code I50.x in any diagnostic position during the period between 2004 and 2024. No exclusion criteria were applied.

Patient data were collected from the Institution’s Health Information System based on digital medical records. The collected data included demographic data (age, sex), laboratory, electrocardiographic and echocardiographic parameters, clinical characteristics (height, weight, comorbidities, symptoms, and signs), as well as data on the classification of chronic heart failure according to ejection fraction and NYHA classification, and medications used.

Kidney function was assessed using the estimated glomerular filtration rate (eGFR) calculated according to the MDRD equation [11]: eGFR = 175 × (serum creatinine [mg/dL])^−1.154^ × (age)^−0.203^ × (0.742 if female) × (1.212 if Black). Based on eGFR values, patients were classified into two categories:

- CKD group with eGFR < 60 mL/min/1.73 m^2^—heart failure patients with chronic kidney disease.
- Non-CKD group with eGFR ≥ 60 mL/min/1.73 m^2^—heart failure patients with preserved renal function [12].

This study was conducted in accordance with the Declaration of Helsinki and approved by the Ethics Committee of the Institute of Cardiovascular Diseases Vojvodina (protocol code 1992-1/6 on 26 December 2024).

### Statistical Analysis

Continuous numerical variables are presented as mean ± standard deviation, whereas continuous non-parametric variables are presented as median with interquartile range (IQR). Categorical variables are presented as frequencies and analyzed using the chi-square test.

For two independent groups, parametric data were analyzed using the Student’s *t*-test, and non-parametric data were analyzed using the Mann–Whitney U test. Pearson’s correlation test was used to assess the relationships between continuous variables. Correlations were evaluated between key laboratory parameters, echocardiographic measurements, and eGFR values to determine the association between renal function and patients’ clinical or echocardiographic characteristics. Statistical significance was set at *p* < 0.05. Statistical analysis was performed using JASP software version 0.19.2.0.

## 3. Results

### 3.1. Study Population

A total of 2227 patients with CHF were included, of whom 1641 (73.69%) had concomitant CKD and 586 (26.31%) had preserved renal function (non-CKD group). The CKD group had a significantly higher prevalence of females (*p* < 0.001). Additionally, patients in the CKD group were considerably older, with an average age of 72.70 ± 9.32 years compared to 65.87 ± 11.00 years in the other group (*p* < 0.001). Within the CKD group, females were also significantly older, averaging 74.04 ± 9.40 years, while females in the other group averaged 69.23 ± 10.28 years (*p* < 0.001). Basic patient characteristics are presented in Table 1.

### 3.2. Clinical Characteristics

**Figure 1:**
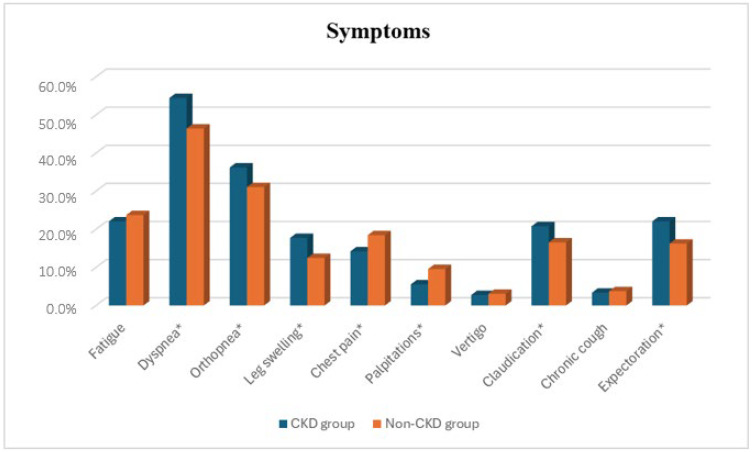
Comparison of symptoms between the two groups (* indicates significant difference).

Dyspnea, orthopnea, leg swelling, claudication, and expectoration were significantly more prevalent in the CKD group (all *p* < 0.05), while chest pain and palpitations were more frequent in non-CKD patients (all *p* < 0.05) (Figure 1). Dyspnea was the most common symptom in both groups, and vertigo was the least common.

Systolic and diastolic blood pressures were higher in non-CKD patients. Physical examination findings, including pulmonary crackles, pleural effusion, mitral regurgitation murmur, carotid murmur, bilateral pretibial edema, and tachypnea, were significantly more frequent in the CKD group (all *p* < 0.05). Cardiac valve surgery and valvular heart disease were more frequent in CKD patients, while hyperlipidemia and hypertension were more prevalent in non-CKD patients (all *p* < 0.05).

The cohort had a high prevalence of HFrEF. There were significantly more male patients in the HFrEF group, while female patients were more prevalent in the HFpEF group (*p* < 0.001). Class IV NYHA was significantly more prevalent in CKD patients, while Classes II and III were more common in non-CKD patients (*p* < 0.001).

In-hospital mortality was 23.9% for the whole heart failure cohort, occurring after a median of 7 days after admission (IQR 3–15; range 0–102 days). Mortality was 2.5-fold higher in patients with CKD compared with those without CKD (28.6% vs. 11.1%; *p* < 0.001).

### 3.3. Laboratory Findings

Patients with CKD exhibited significantly lower hematological parameters, indicating anemia (hemoglobin, mean corpuscular hemoglobin (MCH), mean corpuscular hemoglobin concentration (MCHC), and red blood cells (RBC), all *p* < 0.05, while white blood cell (WBC) counts were higher (*p* < 0.05). Patients with CKD also had significantly higher mean values of C-reactive protein (CRP) and all renal biomarkers (creatinine, urea, and uric acid).

Markers of advanced heart failure, N-terminal pro-brain natriuretic peptide (NT-proBNP), liver enzymes (aspartate aminotransferase (AST), alanine aminotransferase (ALT), and bilirubin as a sign of congestion were higher in CKD patients.

The non-CKD group demonstrated a more pronounced impaired lipid profile, with increased mean low-density lipoprotein (LDL) and total cholesterol and decreased high-density lipoprotein (HDL) (Table 2).

**Figure 2:**
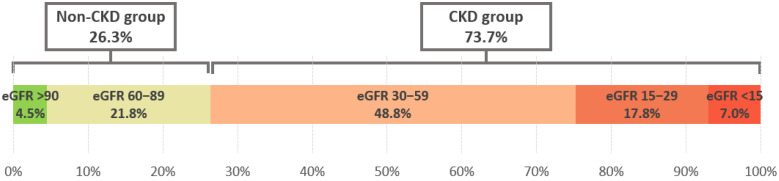
Heart failure patients classified by the levels of eGFR.

The distribution of patients across different levels of eGFR is presented in Figure 2.

The most pronounced negative correlations with eGFR were identified for urea (r = −0.564; *p* < 0.001), uric acid (r = −0.378; *p* < 0.001), and NT-proBNP (r = −0.377; *p* < 0.001), and significant negative associations were also recorded for CRP, AST, ALT, WBC, CK-MB, TSH, and potassium. In contrast, higher eGFR correlated with more favorable hematological and other parameters, including RBC (r = 0.268; *p* < 0.001), hemoglobin (r = 0.257; *p* < 0.001), albumin, and total proteins, while lipid parameters showed weak but statistically significant positive correlations.

### 3.4. Electrocardiography

The CKD group had a lower prevalence of sinus rhythm: 36.99% vs. 50.86%, *p* < 0.001. Supraventricular arrhythmias, ST elevation, and T-wave inversion were more frequent in non-CKD patients. A detailed comparison of ECG changes is displayed in Table 3.

### 3.5. Echocardiography

The non-CKD group exhibited significantly higher mean left ventricular end-diastolic and end-systolic diameters, left ventricular volumes, and stroke volume (*p* < 0.05). Interventricular septum thickness, mitral and aortic valve gradients, RVSP, and E/E′ ratio were higher in patients with CKD (Table 4).

In the echocardiographic evaluation, lower eGFR was associated with more pronounced diastolic dysfunction and greater hemodynamic load, including elevated transmitral and transaortic gradients, higher E/E′ (r = −0.141; *p* < 0.001), thicker left ventricular walls, and increased right ventricular systolic pressure (r = −0.149; *p* < 0.001). Weak but significant positive correlations with eGFR were found for left ventricular volumes and stroke volume, while parameters such as left ventricular ejection fraction, left atrial size, and tricuspid annular plane systolic excursion (TAPSE) did not show a statistically significant association with eGFR.

Mitral (*p* = 0.027) and tricuspid regurgitation (*p* = 0.014) were more prevalent and severe in the CKD group. A statistically significant difference was observed in the distribution of individual categories between the two patient groups (*p* < 0.001). More severe stages of mitral regurgitation were observed in the CKD group.

### 3.6. Therapy

No significant differences were observed in the use of cardiovascular medications, including beta-blockers, angiotensin-converting enzyme inhibitors/angiotensin receptor blockers/angiotensin receptor-neprilysin inhibitors, mineralocorticoid receptor antagonists, diuretics, calcium channel blockers, digoxin, amiodarone, anticoagulants, or statins (Table 5). Antiplatelet drugs were more frequently prescribed in the CKD group (47.5% vs. 42.8%; *p* = 0.050). SGLT2 inhibitors were less frequently prescribed in the CKD group (20.6% vs. 35.2%; *p* < 0.001), although this particular drug use was analyzed only for the last 7 years of the study period, since SGLT2 inhibitors began to be prescribed from 2018 onward.

Renal replacement therapy with hemodialysis was performed in 97 patients, constituting 5.9% of the patients with CKD.

## 4. Discussion

Our study demonstrates that the coexistence of CKD significantly worsens the clinical, laboratory, and echocardiographic profile of patients with CHF, confirming the complex bidirectional interaction between the heart and kidneys, also known as CRS [7,8,9,10]. Although all patients in our study had a diagnosis of CHF based on the selection by using ICD-10 code I50.x, many of the patients were hospitalized due to acute decompensation of CHF, which likely explains the relatively high NT-proBNP levels, advanced NYHA functional class, and reduced left ventricular ejection fraction observed in this cohort.

The high prevalence of CKD in our study can be explained by several factors. This was a large real-world cohort derived from a tertiary referral center, which predominantly manages patients with advanced and more complex heart failure. Additionally, CKD was defined based on a single eGFR measurement at hospital admission, reflecting real-world clinical practice. Acute hemodynamic decompensation, congestion, and neurohumoral activation during hospitalization for heart failure probably contributed to reduced eGFR values in a large number of patients.

This represents a complex pathophysiological entity characterized by the impact of acute and chronic cardiac dysfunction on the deterioration of renal function, corresponding to CRS types 1 and 2. Importantly, the influence of the kidneys on cardiac function is also essential, alongside other comorbidities, as observed in the remaining types of CRS [1,2,3,4,6,13,14]. Hemodynamic alterations resulting from dysfunction of either organ, including increased central venosus pressure and venous congestion, further impair renal perfusion, thereby limiting the kidneys’ ability to perform their physiological roles. Moreover, neurohormonal dysregulation represents an important mechanism in CRS, particularly through activation of the sympathetic nervous system and RAAS. These processes promote vasoconstriction, increased preload and afterload, and subsequent myocardial injury. Metabolic disturbances secondary to renal disfunction, such as uremia, metabolic acidosis, and electrolyte imbalances, may precipitate arrhythmias and further compromise cardiac performance. At the molecular level, CRS reflects a convergence of neurohumoral overactivation, systemic inflammation, endothelial and metabolic dysregulation, oxidative stress, and profibrotic signaling cascades, together with hemodynamic stress, which drive structural and functional deterioration of both cardiac and renal tissues [2,3,4,5,15]. Additionally, pharmacological interventions aimed at supporting one organ may unintentionally impair the other [4]. While the mutual influence between the heart and kidneys is well established, further research is needed to elucidate the consequences of these interactions.

Consistent with previous reports, our study demonstrated that patients with concomitant CKD were older (mean 72.70 ± 9.32 years vs. 65.87 ± 11.00 years, *p* < 0.001) and female sex was more prevalent in this group, reflecting gender-related differences in susceptibility to combined cardiac and renal dysfunction [16,17]. Women were significantly older (74.04 ± 9.40 years) than men (69.23 ± 10.28 years), and there were significantly more male patients in the group with HFrEF, while female patients were more represented in the group with HFpEF (*p* < 0.001). Our findings are in line with previous studies showing that women with heart failure tend to be older, whereas men more frequently develop heart failure with reduced ejection fraction, indicating sex-related differences in disease progression [18].

Clinically, CKD patients exhibited more severe fluid overload, evidenced by a higher prevalence of peripheral edema and pleural effusions, as well as a higher NYHA functional class. These findings align with prior studies reporting that renal impairment amplifies congestion in heart failure [19,20]. In our cohort, dyspnea, orthopnea, peripheral edema, claudication, and expectoration were significantly more frequent among CKD patients, whereas chest pain and palpitations were more prevalent in the non-CKD group. This nuanced symptom distribution is consistent with earlier observations that CKD modifies the heart failure phenotype [19,20].

Our findings are consistent with large multicentric registries and observational studies demonstrating that the presence of CKD is associated with a more severe clinical profile and substantially worse outcomes in patients with heart failure [21,22,23]. Previous studies have demonstrated that renal dysfunction is a strong and independent predictor of mortality in both acute and CHF, reflecting the complex interconnection between heart and kidneys [1]. In line with these data, our results demonstrated that heart failure patients with CKD experienced markedly higher in-hospital mortality compared with those with preserved renal function.

An analysis of previous medical history demonstrated that CKD patients more often had valvular heart disease, pleural effusions, and bilateral edema, whereas non-CKD patients more frequently presented with hyperlipidemia and hypertension. These findings are consistent with prior research reporting that CKD is associated with fluid retention and structural cardiac changes, while traditional cardiovascular risk factors such as dyslipidemia are more pronounced in non-CKD populations [7,8,9,10]. The lower prevalence of hypertension observed in patients with CKD in our study represents a somewhat unexpected result, given the well-established bidirectional interconnection between hypertension and CKD [24,25]. This could be explained by the fact that blood pressure was assessed at the time of admission, while a number of patients selected based on ICD-10 code i50.x could have been in acute decompensation of CHF, when hypotension and low-output states are common, particularly in patients with advanced heart failure and concomitant renal dysfunction [26]. Moreover, patients with CKD in our study were older and had more severe objective signs of heart failure, which may have contributed to lower blood pressure and under-recognition of hypertension as an active diagnosis. Intensive use of diuretics, vasodilators, and neurohormonal therapies in such acute settings may have contributed to lower blood pressure at admission [27]. Similar paradoxical findings regarding blood pressure have been reported in cohorts of patients with advanced heart failure, where hypotension often reflects disease severity rather than an absence of prior hypertension [6].

Laboratory analyses reinforced these findings. CKD patients exhibited higher NT-proBNP and CRP levels, reflecting elevated cardiac stress and systemic inflammation. These observations are in line with previous studies demonstrating that renal impairment is associated with increased inflammatory and profibrotic markers, contributing to adverse cardiac remodeling [7,8,9,10,28,29,30]. Serum creatinine, urea, and uric acid were also significantly elevated in the CKD group, in line with expected markers of renal dysfunction [31]. Interestingly, lipid levels (LDL, HDL, total cholesterol) were higher in non-CKD patients, supporting prior evidence that CKD is associated with altered lipid metabolism [32,33,34].

On echocardiography, CKD patients demonstrated increased interventricular septal thickness, higher E/e’ ratios, elevated right ventricular systolic pressure, and more pronounced mitral and tricuspid regurgitation, suggesting impaired diastolic function, elevated filling pressures, and right heart strain [35,36,37,38,39,40]. Increased interventricular septum thickness indicates left ventricular hypertrophy, consistent with studies by Glassock et al. [37] and Maqbool et al. [38].

Lower eGFR correlated negatively with NT-proBNP, creatinine, and urea while positively correlating with hematologic and protein parameters, emphasizing the integrated pathophysiological link between renal function and cardiac performance [7,8,9,10].

Regarding heart failure phenotype, HFrEF predominated in both groups, while the relative proportion of HFpEF was higher among CKD patients, though not statistically significant. This observation aligns with the literature suggesting that CKD favors HFpEF due to chronic pressure and volume overload. In addition, in our study, CKD was more prevalent among women as was HFpEF, which is also consistent with the existing literature and aligned with our findings demonstrating that HFpEF is more common in the CKD group [6,7,8]. Functional status assessed by NYHA classification revealed that CKD patients more frequently presented with advanced symptoms, corroborating previous studies linking renal impairment to worse functional outcomes in heart failure [7,8,9,10].

Surprisingly, we observed lower use of SGLT2 inhibitors in patients with CKD. These results should be taken with caution, since these drugs were not being used for the first 13 years of the study period. The lower prescription rate of SGLT2 inhibitors in the CKD group reflects several factors. First, during much of the study period, SGLT2 inhibitors were approved primarily for patients with type 2 diabetes and relatively preserved renal function, while their use was discouraged at lower eGFR levels [41,42]. Secondly, concerns regarding volume depletion, acute kidney injury, and electrolyte disturbances in patients with advanced CKD and CHF may have contributed to more cautious prescribing in this population, particularly in older and more fragile patients [43]. Finally, evidence supporting the cardiovascular and renal benefits of SGLT2 inhibitors in patients with heart failure emerged only after the publication of large randomized control trials, such as DAPA-HF [44], EMPEROR-Reduced [45], and EMPEROR-Preserved [46], which likely explains the delayed implementation and lower use of these agents during earlier periods of the study.

Taken together, these results reinforce the prognostic importance of CKD in CHF. Integrating clinical, laboratory, and echocardiographic markers, including E/e’ ratio and RVSP, as well as cardiac remodeling underscores the need for comprehensive evaluation and individualized management strategies addressing both cardiac and renal dysfunction [7,8,9,10,35,36,37,38,47].

### 4.1. Strengths

This study includes a large real-world clinical cohort of more than 2200 CHF patients, providing robust, representative data from a high-volume tertiary care center. Comprehensive integration of clinical characteristics, laboratory biomarkers, and echocardiographic parameters allowed a multidimensional assessment of CKD impact on heart failure severity.

### 4.2. Limitations

Renal function was assessed at hospital admission, and transient worsening of kidney function related to acute heart failure decompensation cannot be fully excluded. CKD was defined solely based on eGFR assessment, as markers of kidney damage like urinary albumin-to-creatinine ratio were not consistently available in this retrospective cohort. Thus, patients with early-stage CKD and preserved eGFR may have been misclassified as non-CKD, potentially leading to an underestimation of the true impact of renal dysfunction. Although diabetes mellitus was recorded as a comorbidity, the specific etiology of CKD, including diabetic nephropathy, could not be reliably determined due to the absence of longitudinal and nephrology-specific data.

Due to the retrospective, cross-sectional design of this study, the temporal relationships between the onset of heart failure and renal dysfunction could not be established with certainty. Therefore, CKD was analyzed as a comorbidity irrespective of whether it preceded or resulted from long-lasting cardiac dysfunction, making it inappropriate for a reliable distinction between specific types of CRS. Finally, the lack of longitudinal follow-up data limited the assessment of adverse events over time, thus limiting the interpretation of causality between CKD and adverse outcomes in heart failure patients.

### 4.3. Future Directions

Future studies should incorporate longitudinal follow-up to assess prognostic impact of combined heart and kidney dysfunction, including morbidity, mortality, and rehospitalization rates. An additional direction for the future research might be focused on the comparison of eGFR at admission and eGFR at discharge, which would evaluate the improvement of kidney function with cardiac recompensation. Prospective research with standardized imaging protocols and detailed biomarker analysis would clarify mechanistic pathways linking CKD to adverse cardiac remodeling. Multivariable modeling to control for confounders could strengthen causal inference. Interventional studies evaluating targeted strategies—such as optimized diuretic therapy, SGLT2 inhibitors, or device-based congestion management—in CHF patients with CKD are warranted. Expanding research to multicenter cohorts would enhance external validity and provide stronger evidence for clinical decision-making.

## 5. Conclusions

In this large real-world cohort of patients with chronic heart failure, the coexistence of chronic kidney disease was associated with a more severe clinical presentation, advanced functional limitation, and a distinct laboratory and echocardiographic profile. The observed associations between declining eGFR and unfavorable clinical, laboratory, and echocardiographic parameters highlight the central role of renal dysfunction as a key modifier of heart failure severity rather than a simple comorbidity. These findings highlight the need for integrated cardiorenal assessment and individualized management strategies in patients with chronic heart failure to improve risk stratification and optimize clinical outcomes.

## Figures and Tables

**Table 1:** Basic characteristics of heart failure patients in the CKD group compared to the non-CKD group.

	CKD Group *n* = 1641	Non-CKD Group *n* = 586	*p*
Study Population			
Age, years	72.70 ± 9.32	65.87 ± 11.00	<0.001
Females, *n* (%)	629 (38.33%)	146 (24.91%)	<0.001
Antropometry			
Body weight, kg	81.22 ± 16.74	83.64 ± 17.65	0.004
Body height, cm	170.47 ± 8.66	172.57 ± 8.39	<0.001
Body mass index, kg/m^2^	27.89 ± 5.15	28.02 ± 5.29	0.611
Symptoms			
Fatigue, *n* (%)	362 (22.06%)	139 (23.72%)	0.409
Dyspnea, *n* (%)	893 (54.42%)	272 (46.42%)	<0.001
Orthopnea, *n* (%)	594 (36.20%)	182 (31.06%)	0.025
Leg swelling, *n* (%)	291 (17.73%)	73 (12.46%)	0.003
Chest pain, *n* (%)	233 (14.20%)	108 (18.43%)	0.015
Palpitations, *n* (%)	91 (5.54%)	56 (9.56%)	<0.001
Vertigo, *n* (%)	45 (2.74%)	18 (3.07%)	0.680
Claudication, *n* (%)	341 (20.78%)	97 (16.55%)	0.027
Chronic cough, *n* (%)	55 (3.35%)	22 (3.75%)	0.647
Expectoration, *n* (%)	362 (22.06%)	95 (16.21%)	0.003
NYHA Classification			
I, *n* (%)	250 (15.57%)	92 (16.23%)	<0.001
II, *n* (%)	55 (3.42%)	59 (10.41%)	
III, *n* (%)	239 (14.88%)	112 (19.75%)	
IV, *n* (%)	1062 (66.13%)	304 (53.62%)	
Clinical Signs			
Pulmonary crackles, *n* (%)	998 (60.82%)	304 (51.88%)	<0.001
Pleural effusion, *n* (%)	304 (18.52%)	73 (12.46%)	<0.001
Mitral regurgitation murmur, *n* (%)	619 (37.72%)	145 (24.74%)	<0.001
Carotid murmur, *n* (%)	223 (13.59%)	51 (8.70%)	0.002
Bilateral pretibial edema, *n* (%)	291 (17.73%)	73 (12.46%)	0.003
Tachypnea, *n* (%)	35 (4.60%)	6 (1.99%)	0.046
Vital Signs			
Systolic blood pressure, mmHg	129.55 ±32.07	133.54 ±29.30	0.011
Diastolic blood pressure, mmHg	75.94 ± 17.46	79.36 ± 16.80	<0.001
Heart rate, beats per minute	91.24 ± 25.73	93.63 ± 26.52	0.065
Past medical history			
Hypertension, *n* (%)	1148 (69.96%)	436 (74.40%)	0.042
Atrial fibrillation, *n* (%)	803 (48.93%)	262 (44.71%)	0.079
Myocardial infarction, *n* (%)	628 (38.27%)	228 (38.91%)	0.785
PCI, *n* (%)	456 (27.79%)	173 (29.52%)	0.423
CABG, *n* (%)	245 (14.93%)	78 (13.31%)	0.339
Valvular heart disease, *n* (%)	1326 (80.80%)	429 (73.21%)	<0.001
Cardiac valve surgery, *n* (%)	194 (11.82%)	47 (8.02%)	0.011
Other cardiac surgery, *n* (%)	52 (3.17%)	18 (3.07%)	0.908
Congenital heart disease, *n* (%)	14 (0.85%)	5 (0.85%)	1.000
Acute rheumatic fever, *n* (%)	24 (1.46%)	7 (1.19%)	0.635
Pacemaker, *n* (%)	169 (10.30%)	47 (8.02%)	0.110
ICD, *n* (%)	38 (2.32%)	16 (2.73%)	0.575
Stroke, *n* (%)	234 (14.26%)	75 (12.80%)	0.380
Diabetes mellitus, *n* (%)	636 (38.76%)	213 (36.35%)	0.303
Hyperlipidemia, *n* (%)	564 (34.37%)	292 (49.83%)	<0.001
COPD/Asthma, *n* (%)	325 (19.80%)	104 (17.75%)	0.278
Heart Failure Type			
HFrEF, *n* (%)	782 (62.1%)	315 (66.9%)	0.114
HFmrEF, *n* (%)	169 (13.4%)	62 (13.2%)	
HFpEF, *n* (%)	309 (24.5%)	94 (19.9%)	
In-hospital mortality	468 (28.6%)	65 (11.1%)	<0.001

PCI—percutaneous coronary intervention; CABG—coronary artery bypass grafting; ICD—implantable cardioverter-defibrillator; COPD—chronic obstructive pulmonary disease; HFmrEF—heart failure with mildly reduced ejection fraction; HFrEF—heart failure with reduced ejection fraction; HFpEF—heart failure with preserved ejection fraction.

**Table 2:** Comparison of laboratory results in heart failure patients in CKD group and non-CKD group.

Laboratory Parameters	CKD Group	Non-CKD Group	*p*
Glucose, mmol/L	8.82 ± 4.62	8.50 ± 4.02	0.151
C-reactive protein, mg/L	47.83 ± 65.59	34.88 ± 58.96	<0.001
Creatinine, µmol/L	183.73 ± 118.93	83.54 ± 15.76	<0.001
BUN, mmol/L	15.14 ± 8.98	7.91 ± 3.75	<0.001
Uric acid, µmol/L	487.93 ± 163.32	363.67 ± 121.57	<0.001
AST, U/L	74.95 ± 209.87	51.96 ± 95.39	0.011
ALT, U/L	76.82 ± 213.75	47.02 ± 115.31	0.002
GGT, U/L	99.62 ± 110.26	81.51 ± 55.11	0.314
Bilirubin, µmol/L	19.79 ± 25.40	17.26 ± 19.39	0.033
Total protein, g/L	69.68 ± 10.20	70.33 ± 20.05	0.344
Albumin, g/L	32.72 ± 6.69	33.13 ± 7.12	0.586
CK-MB, U/L	35.70 ± 58.22	31.31 ± 35.95	0.089
Troponin, ng/L	1637.96 ± 6468.94	1419.22 ± 5654.90	0.569
NT-proBNP, pg/mL	12279.83 ± 9140.16	6882.81 ± 7022.54	<0.001
TSH, μIU/mL	5,21 ± 7.96	3.08 ± 3.01	0.002
Hemoglobin, g/L	120.66 ± 27.92	132.44 ± 24.10	<0.001
Red blood cells, 10^12^/L	4.24 ± 0.78	4.55 ± 0.70	<0.001
MCV, fl	89.62 ± 7.62	89.66 ± 7.39	0.929
MCH, pg	29.20 ± 2.92	29.62 ± 2.83	0.003
MCHC, g/L	287.16 ± 99.50	302.59 ± 87.55	0.001
White blood cells, 10^9^/L	10.20 ± 5.10	9.48 ± 3.89	0.002
Platelets, 10^9^/L	216.46 ± 85.96	224.04 ± 87.80	0.079
Sodium, mmol/L	138.97 ± 6.20	138.96 ± 8.49	0.989
Potassium, mmol/L	4.40 ± 0.65	4.22 ± 0.55	<0.001
Calcium, mmol/L	1.23 ± 0.29	1.23 ± 0.22	0.928
LDL cholesterol, mmol/L	2.59 ± 1.11	2.95 ± 1.48	<0.001
HDL cholesterol, mmol/L	0.98 ± 0.36	1.09 ± 0.40	<0.001
Total cholesterol, mmol/L	4.13 ± 1.46	4.53 ± 1.59	<0.001
Triglycerides, mmol/L	1.47 ± 1.06	1.56 ± 1.14	0.224

BUN—blood urea nitrogen; AST—aspartate aminotransferase; ALT—alanine aminotransferase; GGT—gamma-glutamyl transferase; CK-MB—creatine kinase-MB; NT-proBNP—N-terminal pro-B-type natriuretic peptide; TSH—thyroid-stimulating hormone; MCV—mean corpuscular volume; MCH—mean corpuscular hemoglobin; MCHC—mean corpuscular hemoglobin concentration; LDL cholesterol—low-density lipoprotein cholesterol; HDL cholesterol—high-density lipoprotein cholesterol.

**Table 3:** ECG changes of heart failure patients in CKD group and non-CKD group.

ECG Changes	CKD Group *n* = 1641	Non-CKD Group *n* = 586	*p*
Sinus rhythm, *n* (%)	607 (36.99%)	297 (50.68%)	<0.001
Pacemaker, *n* (%)	169 (10.30%)	47 (8.02%)	0.110
Atrial fibrilation, *n* (%)	803 (48.93%)	262 (44.71%)	0.079
Atrial flutter, *n* (%)	20 (1.22%)	9 (1.54%)	0.561
Other supraventricular arrhythmias, n (%)	34 (2.07%)	25 (4.27%)	0.005
LBBB, *n* (%)	245 (14.93%)	98 (16.72%)	0.302
LAFB, *n* (%)	8 (0.49%)	4 (0.68%)	0.580
ST elevation, *n* (%)	29 (1.77%)	22 (3.75%)	0.006
ST denivelation, *n* (%)	172 (10.48%)	69 (11.78%)	0.387
Inverted T waves, *n* (%)	152 (9.26%)	85 (14.50%)	<0.001

LBBB—left bundle branch block; LAFB—left anterior fascicular block.

**Table 4:** Comparison of echocardiography parameters in heart failure patients in CKD and non-CKD groups.

ECHO Parameters	CKD Group	Non-CKD Group	*p*
LA, mm	45.87 ± 14.04	45.39 ± 17.93	0.563
LVIDD, mm	56.63 ± 9.70	58.15 ± 9.41	0.004
LVIDS, mm	42.93 ± 11.97	44.32 ± 11.47	0.030
IVS, mm	11.93 ± 1.64	11.74 ± 1.69	0.036
PLW, mm	11.89 ± 3.21	11.65 ± 1.52	0.129
LVEDV, mL	146.39 ± 65.58	158.66 ± 70.78	<0.001
LVESV, mL	98.79 ± 59.80	107.78 ± 64.46	0.006
SVLV, mL	47.65 ± 15.32	50.76 ± 15.16	<0.001
LVEF, %	36.68 ± 13.85	35.91 ± 12.87	0.297
E/e’ ratio	18.23 ± 7.96	16.23 ± 8.08	0.005
E V-max, m/s	0.98 ± 0.38	0.92 ± 0.29	0.003
A V-max, m/s	0.89 ± 4.29	0.70 ± 0.27	0.545
Mitral valve maxPG, mmHg	6.73 ± 4.74	5.82 ± 4.08	<0.001
Mitral valve meanPG, mmHg	2.71 ± 2.25	2.37 ± 1.94	0.010
Mitral valve VTI, cm	28.01 ± 12.80	25.93 ± 10.00	0.005
MADd, mm	35.21 ± 4.94	35.34 ± 4.40	0.774
Mitral regurgitation			
Absent, *n* (%)	67 (5.41%)	39 (8.30%)	<0.001
Mild, *n* (%)	563 (45.48%)	240 (51.06%)	
Moderate, *n* (%)	427 (34.49%)	160 (34.04%)	
Severe, *n* (%)	181 (14.62%)	31 (6.60%)	
RVSP, mmHg	47.59 ± 13.31	42.98 ± 13.05	<0.001
Aortic valve maxPG, mmHg	15.32 ± 16.42	12.89 ± 14.25	0.005
Aortic valve meanPG, mmHg	8.57 ± 10.02	7.28 ± 8.94	0.016
Aortic valve VTI, cm	35.11 ± 19.49	31.91 ± 16.10	0.002
Aortic valve annulus, mm	26.82 ± 4.75	26.48 ± 4.58	0.193
Aortic valve leaflets separation, mm	16.07 ± 6.35	17.13 ± 6.52	0.006
Ascending aorta, mm	35.26 ± 6.23	35.07 ± 4.59	0.626

LA—left atrium; LVIDD—left ventricular internal diameter in diastole; LVIDS—left ventricular internal diameter in systole; LVEDV—left ventricular end-diastolic volume; LVESV—left ventricular end-systolic volume; SVLV—stroke volume of the left ventricle; E V-max—peak early diastolic transmitral velocity; A V-max—peak late diastolic transmitral velocity; LVEF—left ventricular ejection fraction; IVS—interventricular septum; PLW—posterior left ventricular wall; Mitral valve maxPG—mitral valve maximum pressure gradient; Mitral valve meanPG—mitral valve mean pressure gradient; Mitral valve VTI—mitral valve velocity time integral; MADd—mitral annular diameter in diastole; E/e’—ratio of early diastolic transmitral velocity to early diastolic mitral annular velocity—left ventricular filling pressure; Aortic valve maxPG—aortic valve maximum pressure gradient; Aortic valve meanPG—aortic valve mean pressure gradient; Aortic valve VTI—aortic valve velocity time integral; RVSP—right ventricular systolic pressure.

**Table 5:** Comparison of medications used in heart failure patients in CKD and non-CKD groups.

Medication	CKD Group	Non-CKD Group	*p*
Beta-blockers, *n* (%)	1107 (67.5%)	394 (67.2%)	0.921
ACEi/ARB/ARNI, *n* (%)	1026 (62.5%)	358 (61.1%)	0.540
MRA, *n* (%)	728 (44.4%)	250 (42.7%)	0.476
SGLT2 inhibitors, *n* (%) *	180 (20.6%)	131 (35.2%)	<0.001
Diuretics, *n* (%)	1133 (69.0%)	403 (68.8%)	0.903
CCB, *n* (%)	56 (3.4%)	16 (2.7%)	0.423
Digoxin, *n* (%)	221 (13.5%)	86 (14.7%)	0.466
Amiodarone, *n* (%)	155 (9.4%)	57 (9.7%)	0.842
Anticoagulants, *n* (%)	642 (39.1%)	247 (42.1%)	0.199
Antiplatelets, *n* (%)	780 (47.5%)	251 (42.8%)	0.050
Statins, *n* (%)	643 (39.2%)	212 (36.2%)	0.199

* analyzed only for the last 7 years of the study period (from 2018 onward). ACEi—angiotensin-converting enzyme inhibitors; ARB—angiotensin receptor blockers; ARNI—angiotensin receptor-neprilysin inhibitors; CCB—calcium channel blockers; MRA—mineralocorticoid receptor antagonists; SGLT2—sodium-glucose cotransporter 2.

## Data Availability

Dataset available on request from the authors.

## Abbreviations

The following abbreviations are used in this manuscript:

CHF	Chronic heart failure
CKD	Chronic kidney disease
CRS	Cardiorenal syndrome
ECG	Electrocardiography
eGFR	Estimated glomerular filtration rate
HFmrEF	Heart failure with mildly reduced ejection fraction
HFpEF	Heart failure with preserved ejection fraction
HFrEF	Heart failure with reduced ejection fraction
NYHA	New York Heart Association
SGLT2	Sodium-glucose cotransporter 2
